# Central adiposity and the overweight risk paradox in aging: follow-up of 130,473 UK Biobank participants

**DOI:** 10.3945/ajcn.116.147157

**Published:** 2017-05-31

**Authors:** Kirsty Bowman, Janice L Atkins, João Delgado, Katarina Kos, George A Kuchel, Alessandro Ble, Luigi Ferrucci, David Melzer

**Affiliations:** 1 Epidemiology and Public Health Group and; 2 Diabetes and Obesity Research Group, Institute of Biomedical and Clinical Sciences, University of Exeter Medical School, Exeter, United Kingdom;; 3 UConn Center on Aging, University of Connecticut Health Center, Farmington, CT; and; 4 National Institute on Aging, Baltimore, MD

**Keywords:** overweight, adiposity, waist-hip ratio, mortality, body mass index, coronary artery disease, UK Biobank, aging, older persons

## Abstract

**Background:** For older groups, being overweight [body mass index (BMI; in kg/m^2^): 25 to <30] is reportedly associated with a lower or similar risk of mortality than being normal weight (BMI: 18.5 to <25). However, this “risk paradox” is partly explained by smoking and disease-associated weight loss. This paradox may also arise from BMI failing to measure fat redistribution to a centralized position in later life.

**Objective:** This study aimed to estimate associations between combined measurements of BMI and waist-to-hip ratio (WHR) with mortality and incident coronary artery disease (CAD).

**Design:** This study followed 130,473 UK Biobank participants aged 60–69 y (baseline 2006–2010) for ≤8.3 y (*n* = 2974 deaths). Current smokers and individuals with recent or disease-associated (e.g., from dementia, heart failure, or cancer) weight loss were excluded, yielding a “healthier agers” group. Survival models were adjusted for age, sex, alcohol intake, smoking history, and educational attainment. Population and sex-specific lower and higher WHR tertiles were <0.91 and ≥0.96 for men and <0.79 and ≥0.85 for women, respectively.

**Results:** Ignoring WHR, the risk of mortality for overweight subjects was similar to that for normal-weight subjects (HR: 1.09; 95% CI: 0.99, 1.19; *P* = 0.066). However, among normal-weight subjects, mortality increased for those with a higher WHR (HR: 1.33; 95% CI: 1.08, 1.65) compared with a lower WHR. Being overweight with a higher WHR was associated with substantial excess mortality (HR: 1.41; 95% CI: 1.25, 1.61) and greatly increased CAD incidence (sub-HR: 1.64; 95% CI: 1.39, 1.93) compared with being normal weight with a lower WHR. There was no interaction between physical activity and BMI plus WHR groups with respect to mortality.

**Conclusions:** For healthier agers (i.e., nonsmokers without disease-associated weight loss), having central adiposity and a BMI corresponding to normal weight or overweight is associated with substantial excess mortality. The claimed BMI-defined overweight risk paradox may result in part from failing to account for central adiposity, rather than reflecting a protective physiologic effect of higher body-fat content in later life.

## INTRODUCTION

The prevalence of BMI-defined overweight and obesity [in kg/m^2^; 25 to <30 and ≥30, respectively] in adults has increased dramatically since 1980, with an estimated 2.1 billion adults affected globally in 2013 (1). Younger and middle-aged adults classified as overweight or class I obese (BMI: 30 to <35) have substantially increased risks for mortality relative to normal-weight individuals (BMI: 18.5 to <25) (2, 3). However, paradoxical associations for persons within the overweight and class I obese BMI categories have been reported for those aged ≥65 y; several meta-analyses and cohort studies show that persons with BMI-defined overweight have mortality risks that are reduced (4, 5) or similar (6–8) to individuals with a normal BMI. Some researchers claim that this paradox may reflect a protective physiologic effect of a slightly higher BMI (9) and challenge the idea that conventional BMI thresholds should be used in older persons (6, 9–11), arguing that this paradox justifies a revision of the current scientific consensus on the health dangers of being overweight. Others have claimed that public health researchers “would rather not talk about” studies that show that being overweight does not always shorten life (12).

Our recent analysis of 955,000 population-representative primary care patients showed that paradoxical overweight and moderate-obesity mortality risks for adults aged 60–84 y were partly explained by confounding from inclusion of smokers and subjects with disease-associated weight loss (13). We found that in “healthier agers” (nonsmokers without disease-associated weight loss), class I obesity was associated with excess mortality and coronary artery disease (CAD) (13); that is, the obesity risk paradox reversed to being nonparadoxical. A further possible bias in the paradoxical associations for BMI-defined overweight (and class I obese) persons may be that BMI does not distinguish between fat and fat-free mass (6, 14). This limitation of BMI may be accentuated in older age groups; after 60 y, fat-free mass decreases and fat mass is redistributed (15, 16) to a more central deposition. Weight loss attributable to the depletion of skeletal mass and height loss during aging may reduce the accuracy of BMI in later life (14). By age 70 y, BMI values are reported to be inflated by 0.7 for men and 1.6 for women (17).

Here we estimated associations between combined BMI and central adiposity measurements with mortality and incident CAD in a large older cohort. The UK Biobank offers an ideal opportunity to estimate these associations in a large sample of “healthier agers” (nonsmokers without disease-associated weight loss) in their seventh decade, in whom the distribution of fat stores to a more central distribution is measured. Waist-to-hip ratio (WHR) is a well-recognized measure of central adiposity (14), includes some adjustment for body shape, and has a relatively weak correlation with BMI (compared with waist circumference alone, which is more strongly correlated with BMI) (7, 17).

## METHODS

Between 2006 and 2010, the UK Biobank recruited >500,000 volunteers across England, Wales, and Scotland; the majority of respondents were aged 40–69 y (range: 37–73 y). At baseline, participants provided self-reports for demographic, socioeconomic, and lifestyle factors. A range of physical measurements, including anthropometrics and blood samples, were also taken at participants’ baseline visit (18, 19). Participants provided informed consent to have their records linked to cancer registrations, hospital admissions, and death registries. The overall response rate was 5.5%, and further cohort details were published previously (18).

For this analysis, we included individuals aged 60–69 y at recruitment. The UK Biobank aimed to recruit participants aged 40–69 y (2247 participants were aged >69 y by the time they were interviewed). We selected only those aged 60–69 y because the obesity paradox has been reported predominantly for this age group and older groups, most female participants were postmenopausal, and fat-mass redistribution to a more central deposition is generally well established in this age group. We excluded participants who were missing BMI (*n* = 1299), waist (*n* = 44), or hip (*n* = 20) measurements. Participants with a BMI <18.5 or ≥35 (*n* = 14,926) were excluded, because the paradox has predominately been reported for those with BMI values within the overweight (25 to <30) and class I obese (30 to <35) ranges.

Participants with missing responses to questions on alcohol intake, educational attainment, or smoking history were excluded (*n* = 5019). To account for subjects with conditions associated with weight loss or altered body distribution, we excluded current smokers and patients who had a previous diagnosis of cancer, heart failure, or dementia (*n* = 38,991), similar to previous reports (13). The chronic condition exclusions were based on our previous analysis (13), which empirically tested the associations of 15 major diagnoses with measured weight loss in a large sample (*n* = 955,000) of primary care patients: cancer, heart failure, and dementia conferred the highest ORs (≥1.5) for measured weight loss. The resulting group consists of “healthier agers” for whom population-level obesity prevention may be relevant. Diagnoses at or before baseline were derived from participants’ self-reports, cancer registries, and hospital admissions (inpatients). We excluded participants whose death dates were not reported (*n* = 1). We excluded the first 2 y of follow-up (*n* = 677) to reduce the effects of reverse causation, whereby underlying diseases are associated with a lower BMI and an increased risk of death. Because previous weight loss is shown to be associated with adverse outcomes, we excluded participants who reported at baseline having lost weight compared with 1 y previously, did not know, or preferred not to answer (*n* = 23,662). The available question on weight loss did not ask about the degree of weight change or whether this weight change was intentional or unintentional. Therefore, our exclusion covered more substantial weight losses as well as minor losses. The remaining sample for analysis thus included 130,473 participants (62,418 men and 68,055 women) (see the flowchart in **Supplemental Figure 1**).

### Exposures

Height, weight, and waist and hip circumferences were measured at the baseline examination after participants removed their shoes and heavy outer clothing. The natural indent was measured once for the waist circumference (the umbilicus was used if the natural indent could not be observed). The hip circumference was recorded once at the widest part of the hips (20). BMI and WHR were derived from the baseline measurements. We used the WHO BMI Classification as follows: normal weight, 18.5 to <25.0; overweight, 25 to <30; and class I obesity, 30 to <35 (21). We categorized WHR by population and sex-specific tertiles as follows: lower WHR, <0.91 for men and <0.79 for women; intermediate WHR, 0.91 to <0.96 for men and 0.79 to <0.85 for women; and higher WHR, ≥0.96 for men and ≥0.85 for women. We also used the proposed WHO binary WHR abdominal obesity cutoff points of >0.85 for women and >0.90 for men (22).

### Covariates

Alcohol intake was defined based on frequency (daily or almost daily, 3–4 times/wk, 1–2 times/wk, 1–3 times/mo, occasionally, and never). Participants were categorized as never or former smokers. The highest level of educational achievement was defined as follows: none; certificate of secondary education; general certificate of secondary education or ordinary level taken at age 15–16 y; advance level, national vocational qualification, higher national diploma, or higher national certificate further education after age 16 y; professional qualification; and college or university degree.

Ethnicity was categorized as white, mixed, Asian, black, Chinese, and other. The mixed category combined the responses of the UK Biobank ethnicity questions of “mixed,” “white and black Caribbean,” “white and black African,” “white and Asian,” and “any other mixed background.”

Participants were categorized as having low, moderate, or high physical activity. This was determined from participants’ responses to frequency and duration of walking, moderate activity, and vigorous activity using the validated International Physical Activity Questionnaire. Total metabolic equivalent minutes of exercise per week were then derived (23).

### Outcomes

Death certificate data were available up to 15 August 2015. These data were collected by the Health and Social Care Information Centre for English and Welsh participants and the Information Services Department for Scottish participants. Data on incident CAD (International Classification of Diseases, 10th revision, codes I20–I25) were available up to 27 February 2015 from Hospital Episode Statistics (England), the Scottish Morbidity Record (Scotland), and the Patient Episode Database for Wales (Wales).

### Statistical analysis

We used Pearson correlation analysis to evaluate the correlations between anthropometric measurements. We used Cox proportional hazards models for categorical mortality analyses (BMI categories, WHR tertiles, and the joint associations of BMI and WHR). Follow-up time for mortality risks was computed from the assessment date until the date of death, or until 15 August 2015 (for survivors). We used Schoenfeld residuals to test the proportional hazards assumption. Competing risks models (accounting for mortality) were used to estimate the association between anthropometric measurements and incident CAD. The follow-up time for incident CAD risks was computed from the assessment date until the date of incident CAD, date of death, or 27 February 2015. Multivariate models were adjusted for age, sex, alcohol intake, smoking history (never or former smoker), and educational attainment. These covariates were chosen because they are in line with previous reports and are similar to our previous analysis (13). We did not adjust for covariates along the casual pathway (i.e., cholesterol and hypertension). Significance was determined at *P* < 0.05. The Akaike information criterion was obtained for each model, with lower values generally indicating improved model fits. Interactions of BMI and WHR tertiles with physical activity, age (groups aged 60–64 y and 65–69 y), smoking history, and sex were also evaluated. Analyses were carried out using Stata statistical software (version 14.0; StataCorp LP).

## RESULTS

### Baseline characteristics

**Table 1** presents the baseline characteristics of the study population (*n* = 130,473). The mean ± SD BMI was 26.9 ± 3.4; 48.9% and 19.5% of participants were classified as overweight and class I obese, respectively. The mean WHR was 0.94 ± 0.06 for men and 0.82 ± 0.07 for women. The correlation between BMI and WHR was 0.58 for men and 0.44 for women. Sex-specific WHR tertiles were defined and derived from the overall study population as follows: lower WHR, <0.91 for men and <0.79 for women; intermediate WHR, 0.91 to <0.96 for men and 0.79 to <0.85 for women; and higher WHR, ≥0.96 for men and ≥0.85 for women. The percentages of participants with lower and higher WHRs were as follows: normal BMI, 57.7% and 12.8%; overweight BMI, 27.1% and 35.2%; and class I obesity, 9.6% and 62.0%, respectively (**Supplemental Table 1**).

**TABLE 1:** Baseline characteristics of UK Biobank participants aged 60–69 y (*n* = 130,473)^1^

Variable	Value
Age, y	64.1 ± 2.8
Sex, F	68,055 (52.2)
BMI, kg/m^2^	26.9 ± 3.4
Normal weight, 18.5 to <25.0	41,369 (31.7)
Overweight, 25.0 to <30.0	63,731 (48.9)
Class I obesity, 30.0 to <35.0	25,373 (19.5)
Waist-to-hip ratio	
Women	0.82 ± 0.07
Men	0.94 ± 0.06
Alcohol intake frequency	
Never	9845 (7.6)
Special occasions only	13,973 (10.7)
1–3 times/mo	12,393 (9.5)
1–2 times/wk	31,348 (24.0)
3–4 times/wk	30,818 (23.6)
Daily or almost daily	32,096 (24.6)
Smoking history	
Never smoker	72,419 (55.5)
Former smoker	58,054 (44.5)
Education	
None	33,157 (25.4)
CSE	2353 (1.8)
GCSE/O-levels	18,627 (14.3)
A-levels/NVQ/HND/HNC	19,318 (14.8)
Professional qualification	20,862 (16.0)
College or university degree	36,156 (27.7)
Diagnosed disease at baseline	
Coronary artery disease	10,115 (7.8)
Type 2 diabetes	6781 (5.2)
Follow-up, y	6.5 ± 0.9

1 Values are means ± SDs or *n* (%). A-levels/NVQ/HND/HNC, advance level/national vocational qualification/higher national diploma/higher national certificate further education after age 16 y; CSE, certificate of secondary education; GCSE/O-levels, general certificate of secondary education/ordinary level taken at age 15–16 y.

### Mortality

Over a maximum follow-up of 8.3 y, 2974 adults died (mean follow-up: 6.5 ± 0.9 y). In survival models adjusted for age, sex, smoking history (never or former smoker), alcohol intake, and educational attainment, individuals categorized as overweight (BMI: 25 to <30) were not at a significantly increased risk of mortality (HR: 1.09; 95% CI: 0.99, 1.19; *P* = 0.066) relative to those categorized as normal weight (BMI: 18.5 to <25). Individuals with class I obesity (BMI: 30 to <35) had a substantially increased mortality risk (HR: 1.27; 95% CI: 1.14, 1.41) compared with normal-weight individuals. Compared with the lowest WHR tertile, the intermediate and highest tertiles were associated with a 12% (HR: 1.12; 95% CI: 1.01, 1.23) and 36% (HR: 1.36; 95% CI: 1.24, 1.49) increased risk of mortality, respectively. These associations with WHR tertiles were attenuated after adjustment for BMI category, with HRs of 1.10 (95% CI: 1.00, 1.21) and 1.32 (95% CI: 1.19, 1.46) for the intermediate and higher WHR tertiles, respectively.

### Combined associations of fat distribution and BMI

**FIGURE 1:**
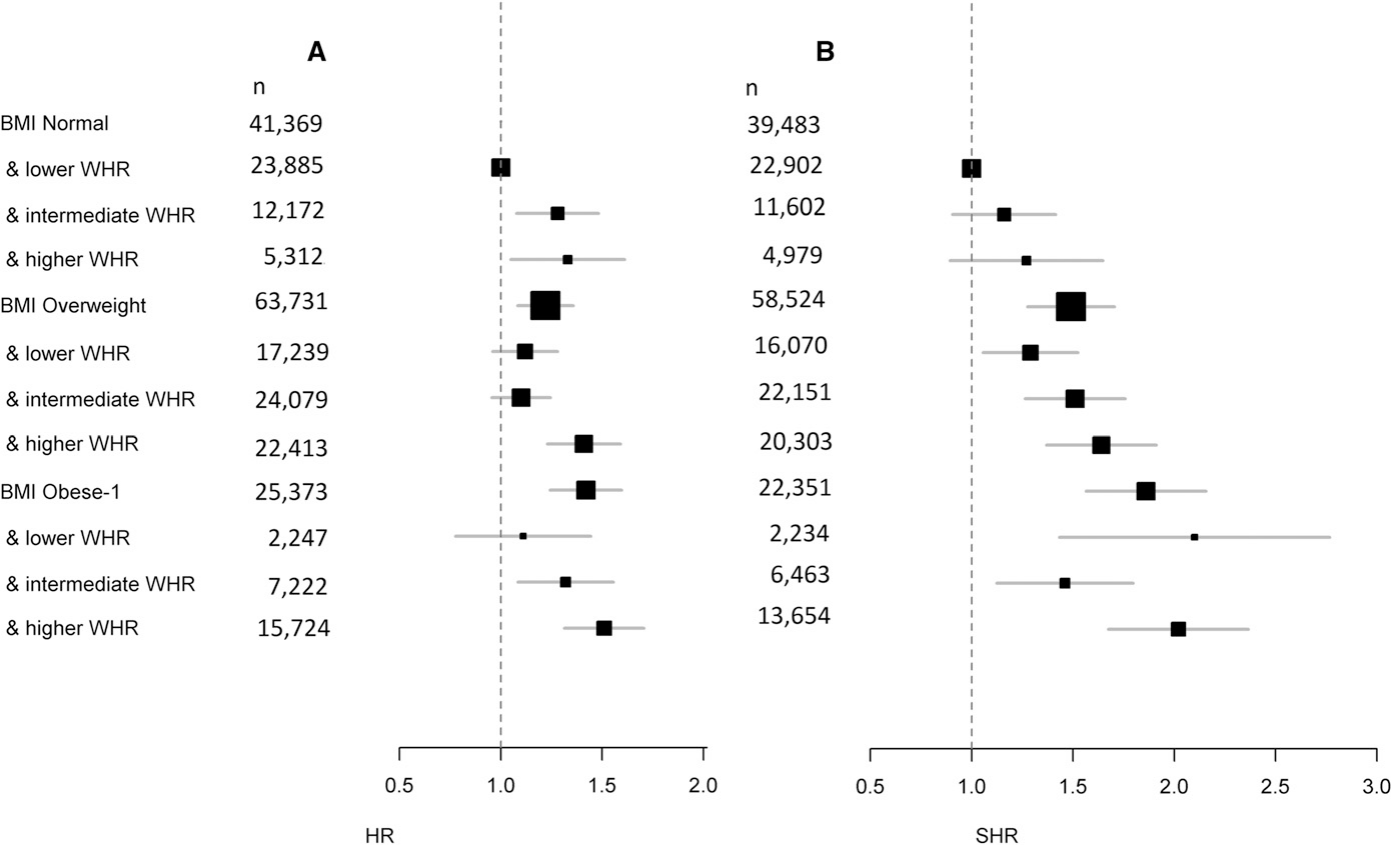
Joint association of BMI categories and WHR tertiles with mortality (A) and CAD (B). (A) Joint associations of BMI categories and WHR tertiles with mortality (*n* = 130,473) for “healthier agers” aged 60–69 y from the UK Biobank. (B) Joint associations of BMI categories and WHR tertiles with incident CAD. Previous cases of CAD were excluded (*n* = 10,115). Competing risk models (accounting for mortality) were used for CAD with SHRs reported. Survival models and competing risk models were adjusted for age, sex, smoking history (never or former smoker), alcohol intake, and educational attainment. Healthier agers included current nonsmokers and individuals without recent or disease-associated (e.g., from dementia, heart failure, or cancer) weight loss. The first 2 y of follow-up were excluded. Values are presented as the number of participants within each category reported for mortality and CAD. The reference group comprised individuals with a normal BMI and lower WHR tertile. WHR was categorized by population sex-specific tertiles as follows: lower WHR, <0.91 for men and <0.79 for women; intermediate WHR, 0.91 to <0.96 for men and 0.79 to <0.85 for women; and higher WHR, ≥0.96 for men and ≥0.85 for women. BMI categories (in kg/m^2^) were as follows: normal weight, 18.5 to <25; overweight, 25 to <30; and class I obesity, 30 to <35. For BMI groupings for BMI and WHR, see Table 1. CAD, coronary artery disease; Obese-1, class I obesity; SHR, sub-HR; WHR, waist-to-hip ratio.

Model fits were improved when both BMI and WHR were included compared with a model with BMI only. **Figure 1A** shows the joint association between BMI category and WHR tertiles (**Supplemental Table 2**). Among normal-weight subjects, those with a higher WHR had increased mortality (HR: 1.33; 95% CI: 1.08, 1.65) compared with those with a lower WHR. In addition, there was an increased risk of 22% and 42% for participants in the overweight (HR: 1.22; 95% CI: 1.09, 1.36) and class I obese (HR: 1.42; 95% CI: 1.25, 1.61) categories overall, respectively, compared with normal-weight adults with a lower WHR. Overweight participants with a higher WHR had a 41% increased risk (HR: 1.41; 95% CI: 1.25, 1.61) and class I obese participants with a higher WHR had a 51% increased risk (HR: 1.51; 95% CI: 1.32, 1.73) relative to the group with a normal BMI and lower WHR.

### CAD

At baseline, there were 10,115 prevalent cases of CAD and these were excluded from the competing risk analysis for incident CAD, yielding 120,358 participants in the competing risk analysis of CAD (incident cases *n* = 1878). Figure 1B shows the joint association between BMI category and WHR tertiles (Supplemental Table 2). Overweight participants with a higher WHR had a 64% increased risk for CAD (sub-HR: 1.64; 95% CI: 1.39, 1.93) relative to normal-weight participants with a lower WHR. There was also an increased risk for incident CAD for overweight participants with a lower or intermediate WHR. Within the group with class I obesity, there was an increased risk for incident CAD for all WHR tertiles relative to the group with a normal BMI and lower WHR tertile.

### Sensitivity analyses

We repeated our main analysis (of the joint association between BMI and WHR tertiles) with age as the underlying time scale, but our results were not substantially changed (**Supplemental Table 3**). We performed an analysis using the proposed WHO WHR abdominal obesity cutoff points (22) of >0.85 for women and >0.90 for men (**Supplemental Table 4**). There was a 22% increased risk of mortality (HR: 1.22; 95% CI: 1.09, 1.37) for overweight participants with a higher WHR and a 42% increased risk (HR: 1.42; 95% CI: 1.26, 1.61) for class I obese participants with a higher WHR relative to normal-weight participants with a lower WHR. We also conducted an analysis using a higher threshold for men with a WHR >1.00, because >75% of the men were classified as centrally obese with the WHO cutoff points. The point estimates were higher for the overweight and class I obese groups with a higher WHR (Supplemental Table 4). There was no significant interaction (*P* < 0.05) between the joint associations of BMI and WHR tertiles with age group (60–64 y and 65–69 y), sex, physical activity, or smoking history (never or former smoker) for mortality. We used a model to estimate how much of the risk between the joint associations of mortality with BMI and WHR tertiles is mediated by diabetes (additionally adjusted for diabetes), and our estimates were not substantially changed (**Supplemental Table 5**). Restricting the analyses to participants who responded that their ethnic background was white/British (*n* = 120,151) only marginally changed the results for BMI and WHR for mortality (Supplemental Table 5); unfortunately, models for other ethnic groups were underpowered. Restricting the analyses to weight-stable participants (*n* = 93,764) did not substantially change the results (Supplemental Table 5).

## DISCUSSION

There is much discussion in the literature about whether being overweight, as defined by BMI, is a risk factor for CAD and all-cause mortality in later life. We aimed to estimate associations between combined BMI and central adiposity measurements with mortality and incident CAD in a large cohort of older, healthier agers. First, we found that models including both BMI and WHR were substantively more informative than models accounting for BMI only. For example, subjects with a normal BMI but higher WHR had increased mortality (HR: 1.33; 95% CI: 1.08, 1.65) compared with those with a lower WHR. We then showed that overweight participants with a higher WHR experienced markedly increased risks for all-cause mortality versus control participants with a normal BMI and lower WHR. There was also an increased risk of incident CAD with increasing WHR tertile within the overweight category. For participants with class I obesity (BMI: 30 to <35), mortality risks were increased and not paradoxical overall, and increasing tertiles of WHR also increased risk within moderate BMI-defined obesity.

It is clear from our data that higher central adiposity in both the normal BMI range and the combination of overweight and central adiposity should be considered a risk factor for clinical risk assessment and public health purposes in healthy agers. Our findings suggest that the reported risk paradox of being overweight in older persons (overweight associated with lower mortality) may be attributable to failure to account for central adiposity, a feature that is not captured by BMI. Controlling or reducing adiposity to increase the chances of aging well (or successful aging) is of particular relevance to our studied group of healthier agers. Our findings therefore do not support the theory that the overweight risk paradox in healthy agers is a real protective physiologic effect (9).

Our study inevitably has limitations, including the use of a volunteer sample, albeit with a wide range of relevant risk exposures (18). The UK Biobank did not aim for population representativeness; because of the wide variation in exposures included in the large sample at baseline, it is likely that the longitudinal risk estimates are relevant for the wider population (24, 25). Our sample was predominately white/British (92%), which may limit the generalizability of our findings to different Caucasian populations. We restricted our analyses to a “healthier agers” group and our risk estimates may be inflated relative to the overall population, owing to fewer competing risk factors (26), although our exclusions were designed to remove confounding and reverse causation. In addition, this group of healthier agers is the main potential target for primary prevention of obesity in later life.

Data on recent weight loss or weight change in the previous 12 mo were based on participants’ self-reports at baseline. It would have been preferable to exclude persons with weight loss over a longer time period and to have an indication of the severity of weight change, but these data were unavailable. Alley et al. (27) reported that the rate of weight loss accelerated during the last 9 y of life for men aged ≥60 y from the Baltimore Longitudinal Study of Aging, so our exclusion of any weight loss in the previous year should have accounted for this effect. Stokes and Preston (28) reported that estimates using baseline BMI may underestimate the association between obesity and mortality, because BMI fluctuations throughout life are not captured; model performance was improved through the use of a person’s maximum attained BMI. Unfortunately, the UK Biobank did not collect data on weight history throughout the life course, so the results from our available BMI data may be underestimates of true effect sizes. Our follow-up period of ≤8.3 y is comparable to other studies, but longer follow-ups may be more informative. Relatively few participants in our class I obese sample had a lower WHR, although many participants in our normal BMI category had intermediate or higher WHRs.

Strengths of our analyses include the large sample of healthier agers and the availability of anthropometric measurements at baseline. In addition, outcomes were ascertained through data from the national death certificate system and hospital records, which are likely to be robust with no loss to follow-up, thus avoiding a common bias in aging cohorts (29).

Our results are difficult to compare with previous work, owing to differing groupings and WHR cutoff points, the inclusion of varying older age ranges, and varied follow-up periods. Our results on the association between WHR tertiles and mortality are in contrast with the nonsignificant associations for the middle and high tertiles reported by Batsis et al. (30) for mortality for adults aged >60 y (*n* = 1569) from NHANES III. This could be attributable to the relatively small sample size and wider age range in the study by Batsis et al., which may have weakened the associations. Our results on the joint association between WHR and BMI categories differ from those of Reis et al. (31), who reported that there was no increased risk of mortality for adults with enlarged WHR tertiles across the normal, overweight, and obese ranges for adults aged 65–100 y, again in a relatively small sample size (*n* = 3748) from NHANES III. Our findings for the combined association between CAD and WHR and BMI categories are also difficult to compare with previous studies because we used the recommended competing-risk model analysis approach, accounting for mortality.

Future work might include a more extensive analysis of alternative measurements of distribution of adiposity, as well as analyses in a wider age range and with longer follow-up. With the accumulation of longer follow-up in UK Biobank, well-powered cause-specific mortality estimates should become feasible. Overall, much work is needed to develop and test effective interventions to limit or reduce excessive adiposity in older groups for whom major gains in healthy aging may thereby be attainable.

In conclusion, in our large sample of 60- to 69-y-old nonsmokers without prior weight loss (or related disease), risk estimation models for mortality combining BMI and WHR were substantially more informative than models with only BMI measurements. The reported BMI-based overweight risk paradox in later life appears to be attributable in part to central adiposity, which is not measured by BMI. Healthier agers (i.e., nonsmokers without weight loss) with higher central adiposity who are in the overweight BMI category have substantial excess mortality and heart disease risk. We also found no evidence of a risk paradox with moderate obesity, but we instead saw overall increases in mortality compared with individuals with a normal BMI and lower WHR. Higher levels of physical activity were an independent protective factor, but we did not find that these negated the effects of overweight or class I obesity. Overall, our findings do not support acceptance of the overweight risk paradox as a real protective physiologic effect in the studied older group.
